# Anesthesia and surgery-induced elevation of CSF sTREM2 is associated with early cognitive dysfunction after thoracoabdominal aortic dissection surgery

**DOI:** 10.1186/s12871-022-01955-4

**Published:** 2022-12-31

**Authors:** Kexin Wang, Xuezhao Cao, Zhe Li, Sidan Liu, Yongjian Zhou, Lili Guo, Pengli Li

**Affiliations:** Department of Anesthesiology, the First Hospital of China Medical University, Shenyang, Liaoning China

**Keywords:** sTREM2, POCD, Aβ_42_, T-tau, P-tau, Aβ_42_/T-tau ratio

## Abstract

**Purpose:**

Soluble triggering receptor expressed on myeloid cells 2 (sTREM2) concentration is increased in cerebrospinal fluid (CSF) in early symptomatic phase of Alzheimer’s disease (AD). This study investigated whether CSF sTREM2 has a relationship with early cognitive dysfunction following surgery in cardiac surgery patients.

**Methods:**

A total of 82 patients undergoing thoracoabdominal aortic replacement were recruited in this study. Neuropsychological testing battery was conducted before and after surgery. Postoperative cognitive dysfunction (POCD) was defined as a Z-score > 1.96 on at least 2 different tests or Telephone Interviews for Cognitive Status-Modified (TICS-M) score < 27. The CSF and serum sTREM2, Aβ_42_, T-tau and P-tau were collected and measured by ELISA on day before surgery and postoperative day 3.

**Results:**

Patients were classified into POCD (*n* = 34) and non-POCD (*n* = 48) groups according to Z-score. Compared to non-POCD group, the levels of CSF sTREM2 (*p* < 0.001) and serum sTREM2 (*p* = 0.001) were significantly higher in POCD group on postoperative day 3. The levels of Aβ_42_ (*p* = 0.005) and Aβ_42_/T-tau ratio (*p* = 0.036) were significantly lower in POCD group on postoperative day 3. Multivariate logistic regression analysis revealed that higher value of postoperative CSF sTREM2 (odds ratio: 1.06, 95% confidence interval: 1.02–1.11, *p* = 0.009), age (OR: 1.15, 95%CI: 1.03–1.28, *p* = 0.014) and POD duration (OR: 2.47, 95%CI: 1.15–5.29, *p* = 0.02) were the risk factors of POCD.

**Conclusion:**

This study indicates that anesthesia and surgery-induced elevation of CSF sTREM2 is associated with an increased risk of early cognitive dysfunction following surgery.

## Introduction

Postoperative cognitive dysfunction (POCD) refers to disorders affecting orientation, attention, perception, consciousness, and judgment that develop after cardiac and non-cardiac surgery [1]. It is a common central nervous system complication of anesthesia and surgery, especially in patients undergoing cardiac surgery with cardiopulmonary bypass (CPB) [2]. POCD affects up to 65% of cardiac surgery patients at hospital discharge [2], resulting in delayed recovery, prolonged length of hospital stay, and an increased risk of disability and mortality [3, 4]. The International Study of POCD estimated the overall incidence of POCD at 19.5% at 1 week and 9.6% after 3 months in the non-cardiac surgery patients [5]. Although the neurobiological basis of POCD remains unknown, major risk factors, such as advanced age, poor education, preexisting cognitive impairment, severity of coexisting illness, duration of anesthesia, respiratory complications and second operation, have been identified [6]. Of these, age has been increasingly reported as the most prominent risk factor for the development of POCD [6]. Owing to elusive pathophysiology and neuropsychological testing battery requirements, POCD diagnosis is usually delayed. Thus, it is necessary to identify reliable and convenient biomarkers for diagnosis and prognosis of POCD.

There is no standard biomarker for POCD prediction and risk stratification. A study by Rasmussen et al. found that there was no relationship between neuron-specific enolase (NSE) or S100β and cognitive decline in elderly patients undergoing abdominal surgery [7]. Similarly, Linstedt et al. revealed an association of S100β but not NSE with early cognitive impairment in the patients undergoing noncardiac surgery [8]. Multiple studies found that the level of tau was increased in the patients who developed POCD [9, 10]. There was significant relationship between perioperative Aβ_42_/T-tau ratio and cognition dysfunction [11, 12]. Thus, it is necessary to find a biomarker with high specificity and sensitivity for POCD predication.

Neuroinflammation plays a crucial role in the progression of POCD [13]. Multiple evidence demonstrated that triggering receptor expressed on myeloid cells 2 (TREM2) inhibited neuroinflammatory responses by repression of microglia-mediated cytokine production [14]. The extracellular domain of TREM2 can be shed by ADAM10/17 cleavage and released in its soluble form into the interstitial fluid of the brain [15–17]. Increased soluble TREM2 (sTREM2) in cerebrospinal fluid (CSF) is related to cognitive impairment in Alzheimer’s disease (AD) patients [18, 19]. These data suggest that elevated sTREM2 may involve in early cognitive dysfunction. Therefore, investigating the association between sTREM2 and POCD in surgical subjects might be conducive.

There are no sensitive and specific biomarkers as predictors of POCD. A study by Ferri et al. has indicated the potential application of sTREM2 as a biomarker of cognitive impairment [20]. However, whether sTREM2 can serve as a predictor of POCD in surgical subjects remains unclear. To improve diagnosis and treatment of POCD, the primary objective of this study was to identify the relationship between CSF sTREM2 and POCD.

## Materials and methods

### Participants

This study protocol was approved by the Ethics Committee of the First Hospital of China Medical University (2020–302-2) and registered in Chinese Clinical Trial Registry Centre (Registration No. ChiCTR2000038634). Between August 2020 and October 2021, the patients with thoracoabdominal aortic dissection were recruited in this study. Informed consent was obtained from all patients. Inclusion criteria was as follows: (1) The age ranges from 30 to 70; (2) The patients undergoing thoracoabdominal aortic surgery under general anesthesia; (3) Lumbar puncture for CSF drainage by L3–4 was performed to prevent paraplegia. Exclusion criteria: (1) Language/communication difficulties; (2) Dementia disorders due to other causes; (3) Contraindications to lumbar puncture; (4) A history of cognitive, psychiatric, or neurological illness; (5) Unavailable for neuropsychological test or refused to participate; (6) MMSE score < 24; (7) A history of cardiovascular surgery or craniotomy.

### Anesthesia management and surgical procedures

The lumbar puncture was performed and a catheter was inserted into the subarachnoid space for CSF drainage to confer neurological protection on day before surgery. All participants received routine monitoring, including blood pressure, electrocardiogram, end-tidal carbon dioxide (ETCO_2_), oxygen saturation, depth of anesthesia by BIS, and temperature on surgery day. Local cerebral oxygen saturation (rSO_2_) was also monitored. Midazolam, etomidate, sufentanil, and cisatracurium were administrated for anesthesia induction. Approximately 5 minutes later, the patients were intubated and mechanically ventilated to maintain ETCO_2_ between 35 and 45 mmHg. Anesthesia was maintained via propofol, sevoflurane, sufentanil, and cisatracurium to adjust BIS values between 40 and 60. All participants underwent CPB with deep hypothermic circulatory arrest. Bilateral anterograde cerebral perfusion was performed to maintain rSO_2_ ≥ 50% or within 20% of preoperative value during deep hypothermic circulatory arrest.

### Neuropsychological tests

Neuropsychological testing battery was performed to evaluate cognitive function on day before surgery and postoperative days 3 and 7. The tests consisted of Mini-mental State Examination (MMSE), symbol digit modalities test (SDMT), digit span forward test (DSFT) and digit span backward test (DSBT), Rey auditory verbal learning test (RAVLT), Stroop color-word test (SCWT) and the confusion assessment method - intensive care unit (CAM-ICU). SCWT consists of three tests: a word test (SCWT-A), a color test (SCWT-B), and a word-color interference test (SCWT-C). Telephone Interviews for Cognitive Status-Modified (TICS-M) and score of activities of daily living (ADLs) were conducted to reduce the loss-to-follow-up rate for discharged patients in postoperative months 1, 3 and 6.

### CSF and serum collection and storage

CSF (5 ml) and blood (5 ml) samples were collected (8:00–12:00 a.m.) on day before surgery and postoperative day 3. The CSF samples were centrifuged at 2000 g for 10 minutes and the blood samples were centrifuged at 1500 g for 5 minutes at room temperature. The supernatants of CSF and serum were transferred into EP tubes and then stored at -80 °C pending biochemical analysis.

### Measurement

Enzyme-linked immunosorbent assay (ELISA) was used to determine sTREM2 (Human TREM2 ELISA kit; Abcam, no. Ab224881), Aβ_42_ (Human Aβ_42_ ELISA kit; JL11012), T-tau (Human T-tau ELISA kit; JL46220) and P-tau (Human P-tau ELISA kit; JL46213). The samples were diluted as recommended by the manufacturer, and a standard curve was used to calculate the concentrations. Briefly, diluted standards, the samples, and antibody were added to the wells. Then, a peroxidase substrate tetramethyl benzidine (TMB) was added for color development. The OD450 was determined using microplate reader and the concentration of the tested substances was calculated by using software ELISACalc.

### Calculation of POCD and POD

POCD was defined as a Z-score > 1.96 on at least 2 different tests on postoperative days 3 or 7, or TICS-M score < 27 in postoperative 1, 3 or 6 months [21–24]. Briefly, Z-score was calculated by the following way: ([change score]_individual_-[mean change score])/(standard deviation change score_total_) [25, 26]. The diagnosis of postoperative delirium (POD) employed CAM-ICU on postoperative days 3 and 7 [27, 28].

### Statistical analysis

Sample size calculation was performed using PASS software, with 80% power, 0.05 alpha, and the expected OR value is 4. Considering a 20% dropout rate, a total of 100 patients was required.

Data were analyzed with IBM SPSS Statistics Version 26 and submitted to Shapiro-Wilk test for normality evaluation. Continuous variables were expressed as means±SD, medians and interquartile ranges (IQRs) and tested using t-tests or Mann-Whitney U tests. Categorical variables were expressed as percentage and tested using Chi-square tests. Comparison among groups was performed using independent t test for biomarker levels and Mann-Whitney U test for neuropsychological tests. Comparison within groups was performed using paired t test for biomarker and Friedman test for neuropsychological tests. Correlations were analyzed with Spearman correlation test, and multiple comparisons were applied with Bonferroni adjustment method. Univariate and multivariate logistic regressions analyses were performed to identify the risk factors of POCD. A *p*-value≤0.05 was considered as statistically significant. The figures were made using GraphPad Prism 8.0 software.

## Result

### Patient characteristics

Characteristics and clinical data of patients were summarized in Table 1. A total of 82 patients undergoing thoracoabdominal aortic replacement were enrolled in this trial. The patients were classified into POCD group (*n* = 34) and non-POCD group (*n* = 48) according to Z-score method. The incidence of POCD was 29.3 and 24.4%, respectively on postoperative days 3 and 7. In 1, 3 and 6 months, the incidence of POCD was 22.0, 17.1 and 11.0%, respectively. The patients who developed POCD were significantly older (*p* < 0.001) and had shorter duration of education (*p* = 0.02) than those without POCD. In addition, a higher incidence of POD and longer POD duration were observed in the patients with POCD (*p* < 0.001 and *p* < 0.001, respectively).

**Table 1:** Demographics characteristics and clinical data of the study samples

Parameters	POCD (***n*** = 34)	Non-POCD (***n*** = 48)	***p***-value
Age (yr), mean ± SD	60.2 ± 6.8	44.1 ± 8.1	< 0.001^**a**^
Male, n (%)	24 (70.6)	37 (77.1)	0.51
BMI (kg/m^2^), mean ± SD	25.4 ± 3.2	25.6 ± 3.3	0.86
Duration of education (yr), median (IQR)	8 (3)	9 (2)	0.02^**b**^
ASA classification, n (%)			0.77
III	18 (52.9)	27 (56.3)	
IV	16 (47.1)	21 (43.7)	
Smoking, n (%)	17 (50.0)	21 (31.8)	0.58
Drinking, n (%)	14 (41.2)	13 (27.1)	0.18
Hypertension, n (%)	31 (91.2)	42 (87.5)	0.60
Diabetes, n (%)	5 (14.7)	6 (12.5)	0.77
Sleep duration (h), median (IQR)	7 (2)	7 (1)	0.10
Duration of surgery (h), median (IQR)	6.5 (0.9)	6.5 (1.7)	0.58
CPB duration (min), mean ± SD	153.4 ± 25.5	153.3 ± 29.4	0.98
Aortic cross-clamp duration (min), mean ± SD	103.8 ± 26.00	108.8 ± 23.2	0.37
Circulatory arrest duration (min), median (IQR)	17 (4)	16 (4)	0.87
Perfusion pressure (mmHg), median (IQR)	180 (30)	180 (30)	0.85
Perfusion volume (ml), median (IQR)	1950 (1200)	2000 (800)	0.64
Deep hypothermic duration (min), mean ± SD	54.7 ± 7.8	51.4 ± 8.6	0.08
Minimum temperature (°C), median (IQR)	21.7 (0.9)	21.4 (1.5)	0.38
Midazolam (mg), median (IQR)	5 (4.3)	5 (4.8)	0.98
Sufentanil (ug), median (IQR)	70 (40)	70 (35)	0.55
Propofol (mg), mean ± SD	1221.3 ± 493.8	1250.5 ± 509.6	0.80
Mechanical ventilation time (h), median (IQR)	47 (32)	38 (30)	0.08
ICU length of stay (d), median (IQR)	7.5(4.0)	6.0 (4.8)	0.08
*POD*, n (%)	23 (67.6)	9 (18.8)	< 0.001^**c**^
POD duration (d), median (IQR)	2 (3)	0 (0)	< 0.001^**d**^
Length of hospital stay (d), median (IQR)	24 (9)	22 (13)	0.41

Data are shown as count n (%), median and interquartile range (IQR) or mean ± SD. *POCD* postoperative cognitive dysfunction, *BMI* Body Mass Index, *ASA* American Society of Anesthesiologists, *CPB* cardiopulmonary bypass, *ICU* intensive care unit. Duration of deep hypothermic: nasopharyngeal temperature < 28 °C; *POD*, postoperative delirium; ^**a**^ t test; ^**b,d**^ Mann-Whitney U test; ^**c**^ χ2 test.

### Neuropsychological tests score

The neuropsychological test battery results were presented in Tables 2 and 3. There were no significant differences for preoperative neuropsychological test battery scores in POCD and non-POCD groups. Postoperative test battery scores were significantly decreased compared with group-matched baseline. Furthermore, the lower scores and longer time consumption were found in POCD group compared to non-POCD group (Table 2). The lower TICS-M and ADL scores were also observed in POCD group in 1, 3 and 6 months postoperatively (Table 3).

**Table 2:** Score for neuropsychological tests

Tests	Baseline	Postoperative day 3	Postoperative day 7
DSFT (n)			
POCD	8 (1)	3.5 (3) ^**,**a**^	4 (2) ^**,**a**^
Non-POCD	8 (1)	6 (1) ^**a**^	7 (1) ^**a**^
DSBT (n)			
POCD	6 (1)	3 (0) ^**,**a**^	3 (1) ^**,**a**^
Non-POCD	5 (2)	4 (1) ^**a**^	4 (1) ^**a**^
RAVLT (n)			
POCD	27.5 (6)	14.5 (6) ^**,**a**^	18 (3) ^**,**a**^
Non-POCD	30 (6)	21.5 (8) ^**a**^	25 (7) ^**a**^
SDMT (n)			
POCD	42.5 (15)	8.5 (5) ^**,**a**^	16.5 (9)^**,**a**^
Non-POCD	39 (9)	20.5 (15) ^**a**^	26 (10) ^**a**^
SCWT-A (s)			
POCD	46 (9)	88 (24) ^**,**a**^	86.5 (23) ^*,**a**^
Non-POCD	44.5 (9)	67 (15) ^**a**^	53.5 (11) ^**a**^
SCWT-B (s)			
POCD	42.5 (15)	93.5 (17) ^**,**a**^	87 (20) ^**,**a**^
Non-POCD	45 (15)	67.5 (11) ^**a**^	59 (13) ^**a**^
SCWT-C (s)			
POCD	51.5 (16)	130.5 (59) ^**,**a**^	116 (47) ^**,**a**^
Non-POCD	54 (10)	88.5 (22) ^**a**^	73 (12) ^**a**^

Test results are either number of correct answer (n) or time consumption (s). Data are presented as median (IQR); *POCD* postoperative cognitive dysfunctionm, *DSFT*, Digit Span Forward Test, *DSBT* Digit Span Backward Test, *RAVLT* Rey Auditory Verbal Learning Test, *SDMT* Symbol Digit Modalities Test, *SCWT* Stroop Color Word Test, * *p* < 0.05 compared with the non-POCD group (Mann-Whitney U test); ** *p* < 0.001 compared with the non-POCD group (Mann-Whitney U test); ^**a**^ *p* < 0.001 compared with baseline (Friedman test).

**Table 3:** The TICS-M score and ADL score between the two group patients

Tests	Postoperative month 1	Postoperative month 3	Postoperative month 6
TICS-M			
POCD	26 (6) ^**^	29 (7) ^**^	35 (11) ^*^
Non-POCD	34 (4)	37 (2)	39 (2)
ADLs			
POCD	33 (20) ^**^	53 (16) ^**^	75 (15) ^*^
Non-POCD	45 (10)	75 (10)	80 (5)

Data are presented as median (IQR); *POCD* postoperative cognitive dysfunction, *TICS-M* Telephone Interview for Cognitive Status-Modified, *ADLs* score of Activities of Daily Living, * *p* < 0.05 compared with the non-POCD group (Mann-Whitney U test); ** *p* < 0.001 compared with the non-POCD group (Mann-Whitney U test).

### CSF and serum concentrations of biomarkers

**Fig. 1:**
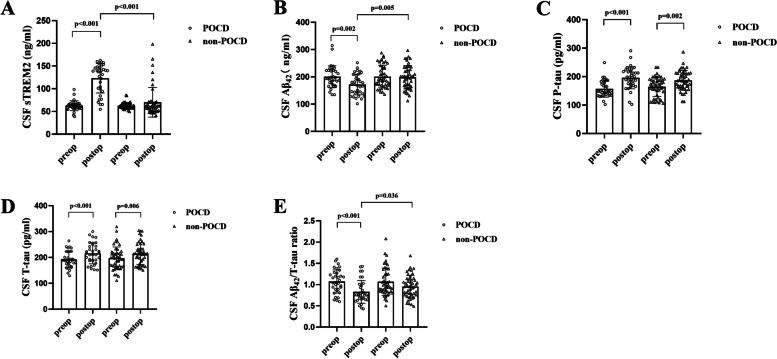
The levels of CSF biomarkers. The levels of CSF sTREM2, Aβ_42_, P-tau, T-tau, and Aβ_42_/T-tau ratio in POCD and non-POCD group. **(A)** CSF sTREM2 was significantly increased on postoperative day 3 and the higher level of CSF sTREM2 was observed in POCD group than that in non-POCD group (*p* < 0.001 and p < 0.001, respectively). **(B)** The lower level of CSF Aβ_42_ was observed in POCD group than that in non-POCD group (*p* = 0.005). **(C)** CSF P-tau was significantly increased in both groups (*p* < 0.05 and p < 0.05, respectively) on postoperative day 3. **(D)** Postoperative CSF T-tau was significantly increased in both groups (p < 0.05 and p < 0.05, respectively). **(E)** Lower CSF Aβ_42_/T-tau ratio was observed in POCD group compared to non-POCD group on postoperative day 3 (*p* = 0.036). Abbreviation: CSF, cerebrospinal fluid; sTREM2, soluble triggering receptor expressed on myeloid cells 2; Aβ_42_, β-amyloid 42; P-tau, phosphorylated tau; T-tau, total tau; POCD, postoperative cognitive dysfunction

The levels of CSF biomarkers were presented in Fig. 1. CSF sTREM2 was significantly increased compared with baseline in POCD group (*p* < 0.001) and the higher level of CSF sTREM2 was observed in POCD group than that in non-POCD group on postoperative day 3 (*p* < 0.001; Fig. 1A). Postoperative CSF Aβ_42_ was significantly decreased in POCD group (*p =* 0.002). Furthermore, the lower level of CSF Aβ_42_ was observed in POCD group than that in non-POCD group (*p =* 0.005; Fig. 1B ). Postoperative CSF P-tau and T-tau were significantly increased in both POCD and non-POCD groups (*p* < 0.05 and *p* < 0.05, respectively). However, no significant differences were found for postoperative CSF P-tau and T-tau between POCD group and non-POCD group (Fig. 1C and Fig. 1)D. Postoperative CSF Aβ_42_/T-tau ratio was significantly decreased in POCD group (*p* < 0.001). Additionally, lower CSF Aβ_42_/T-tau ratio was observed in POCD group compared to non-POCD group on postoperative day 3 (*p* = 0.036; Fig. 1E).

**Fig. 2:**
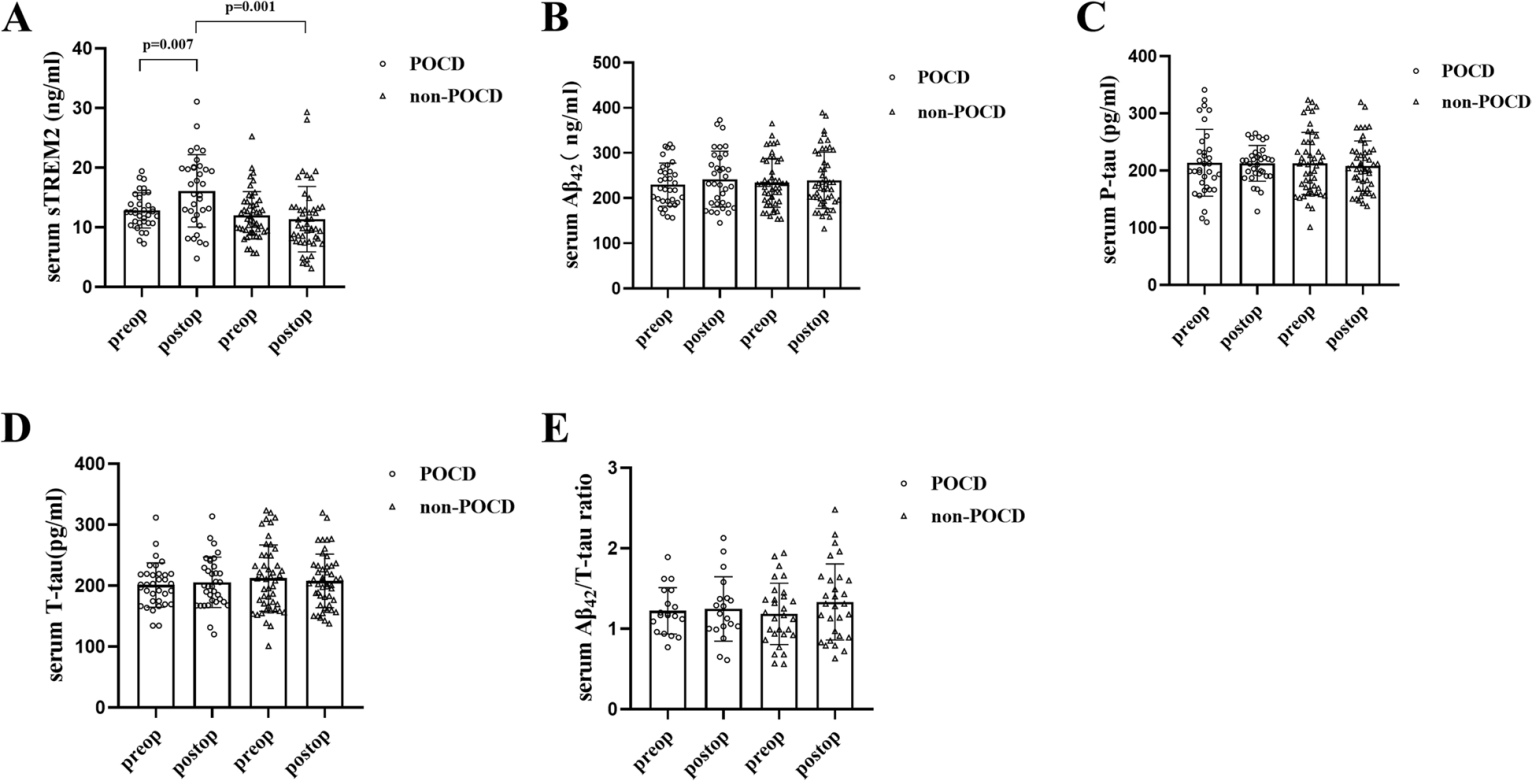
The levels of serum biomarkers. The levels of serum sTREM2, Aβ_42_, P-tau, T-tau, and Aβ_42_/T-tau ratio in POCD and non-POCD group. **(A)** Postoperative serum sTREM2 was significantly increased in POCD group (*p* = 0.007) and the higher level of serum sTREM2 was observed in POCD group than that in non-POCD group (*p* = 0.001). **(B-D)** No significant differences were observed for serum Aβ_42_, P-tau and T-tau between POCD and non-POCD groups on postoperative day 3. **(E)** There was no significant difference in Aβ_42_/T-tau ratio between POCD and non-POCD groups on postoperative day 3. Abbreviation: CSF, cerebrospinal fluid; sTREM2, soluble triggering receptor expressed on myeloid cells 2; Aβ_42_, β-amyloid 42; P-tau, phosphorylated tau; T-tau, total tau; POCD, postoperative cognitive dysfunction

The levels of serum biomarkers were presented in Fig. 2. Postoperative serum sTREM2 was significantly increased in POCD group (*p* = 0.007) and the higher level of serum sTREM2 was observed in POCD group than that in non-POCD group (*p =* 0.001; Fig. 2A). There were no significant differences in serum Aβ_42_, P-tau, T-tau and Aβ_42_/T-tau ratio on postoperative day 3 compared to baseline in both groups. Additionally, no significant differences in serum Aβ_42_, P-tau, T-tau and Aβ_42_/T-tau ratio were observed between POCD and non-POCD groups on postoperative day 3 (Fig. 2B-E).

Analyses of the correlation of postoperative CSF sTREM2 values and other factors were showed in Table 4. Postoperative CSF sTREM2 was positively correlated with postoperative serum sTREM2 (Spearman *r* = 0.381, *p* < 0.05) and age (Spearman *r* = 0.459, *p <* 0.05) and time taken (*p* < 0.05), and negatively correlated with the correct numbers of postoperative neuropsychological tests (*p* < 0.05) and score of TICS-M (*p* < 0.05).

**Table 4:** Factors associated with postoperative CSF sTREM2 values

Factors	Correlation Coefficient	***p***-value
CSF Aβ_42_ postop (ng/ml)	−0.219	>0.05
CSF P-tau postop (pg/ml)	0.204	>0.05
CSF T-tau postop (pg/ml)	−0.040	>0.05
CSF Aβ_42_/T-tau ratio postop	−0.181	>0.05
CSF sTREM2 preop (pg/ml)	0.055	>0.05
Serum sTREM2 postop (pg/ml)	0.381	**<0.05**
Age (yr)	0.459	**<0.05**
Duration of education (yr)	−0.271	>0.05
Duration of POD (d)	0.442	>0.05
DSFT preop (n)	0.004	>0.05
DSFT postop 3d (n)	−0.377	**<0.05**
DSFT postop 7d (n)	−0.403	**<0.05**
DSBT preop (n)	0.066	>0.05
DSBT postop 3d (n)	−0.356	**<0.05**
DSBT postop 7d (n)	−0.403	**<0.05**
RAVLT preop (n)	−0.288	>0.05
RAVLT postop 3d (n)	−0.383	**<0.05**
RAVLT postop 7d (n)	−0.513	**<0.05**
SDMT preop (n)	−0.019	>0.05
SDMT postop 3d (n)	−0.510	**<0.05**
SDMT postop 7d (n)	−0.457	**<0.05**
SCWT-A preop (s)	0.174	>0.05
SCWT-A postop 3d (s)	0.391	**<0.05**
SCWT-A postop 7d (s)	0.524	**<0.05**
SCWT-B preop (s)	0.025	>0.05
SCWT-B postop 3d (s)	0.498	**<0.05**
SCWT-B postop 7d (s)	0.557	**<0.05**
SCWT-C preop (s)	0.001	>0.05
SCWT-C postop 3d (s)	0.387	**<0.05**
SCWT-C postop 7d (s)	0.427	**<0.05**
TICS-M postop 1 m	−0.393	**<0.05**
TICS-M postop 3 m	−0.480	**<0.05**
TICS-M postop 6 m	−0.373	**<0.05**

Correlation coefficients represent non-parametric Spearman’s Rho correlation coefficients; Test results are either number of correct answer (n) or time consumption (s); *P* value was followed by Bonferroni correction. *CSF* cerebrospinal fluid, *sTREM2* soluble triggering receptor expressed on myeloid cells 2, *Aβ*_*42*_ β-amyloid 42, *P-tau* phosphorylated tau, *T-tau* total tau; preop, preoperative, *postop 3d* postoperative day 3, *postop 7d* postoperative day 7, *postop 1 m* 1 month after surgery, *postop 3 m* 3 month after surgery, *postop 6 m* 6 month after surgery, *POD* postoperative delirium, *DSFT* Digit Span Forward Test, *DSBT* Digit Span Backward Test, *RAVLT* Rey Auditory Verbal Learning Test, *SDMT* Symbol Digit Modalities Test, *SCWT* Stroop Color Word Test, *TICS-M* Telephone Interviews for Cognitive Status-Modified.

### Univariate and multivariate logistic regression analysis

Seven potential risk factors of POCD were identified by univariate logistic regression analysis, including postoperative CSF sTREM2, Aβ_42_, Aβ_42_/T-tau ratio, serum sTREM2, age, duration of POD and education (Table 5). The relationship between POCD and risk factors was determined by multivariable logistic regression analysis. After adjustment of potential factors, postoperative CSF sTREM2 (odds ratio: 1.06, 95% confidence interval: 1.02–1.11, *p* = 0.009), age (OR: 1.15, 95%CI: 1.03–1.28, *p* = 0.014) and POD duration (OR: 2.47, 95%CI: 1.15–5.29, *p* = 0.020) were the risk factors of POCD (Table 5).

**Table 5:** The logistic regression of POCD-associated risk factors

parameters	Univariate		Multivariate	
	OR (95%CI)	***p***-value	OR (95%CI)	***p***-value
CSF sTREM2 postop (pg/ml)	1.04 (1.03–1.06)	< 0.001	1.06 (1.02–1.11)	0.009
CSF Aβ_42_ postop (ng/ml)	0.98 (0.97–1.00)	0.007	0.98 (0.96–1.01)	0.135
CSF P-tau postop (pg/ml)	1.01 (0.99–1.02)	0.27		
CSF T-tau postop (pg/ml)	1.00 (0.99–1.01)	0.92		
CSF Aβ_42_/T-tau ratio postop	0.15 (0.03–0.92)	0.041	2.61 (0.02–6.99)	0.701
Serum sTREM2 postop (pg/ml)	1.15 (1.06–1.26)	< 0.001	1.02 (0.87–1.18)	0.841
Serum Aβ_42_ postop (ng/ml)	1.00 (0.99–1.01)	0.85		
Serum P-tau postop (pg/ml)	1.00 (0.99–1.02)	0.58		
Serum T-tau postop (pg/ml)	0.99 (0.98–1.01)	0.36		
Serum Aβ_42_/T-tau ratio postop	1.61 (0.54–4.79)	0.39		
Age (yr)	1.39 (1.19–1.62)	< 0.001	1.15 (1.03–1.28)	0.014
Education (yr)	0.71 (0.52–0.97)	0.03	0.91 (0.54–1.53)	0.731
POD duration (d)	3.01 (1.88–4.81)	< 0.001	2.47 (1.15–5.29)	0.020

*CI* confidence interval, *OR* odds ratio, *POCD* postoperative cognitive dysfunction, *CSF* cerebrospinal fluid, *sTREM2* soluble triggering receptor expressed on myeloid cells 2, *Aβ*_*42*_ β-amyloid 42, *P-tau* phosphorylated tau, *T-tau* total tau, *postop* postoperative, *POD* postoperative delirium.

## Discussion

Our study investigated the relationship between sTREM2 and POCD in the patients undergoing thoracoabdominal aortic surgery. This study demonstrated that the level of CSF sTREM2 was significantly increased in the subjects who developed POCD on postoperative day 3. Elevated CSF sTREM2 was associated with an increased risk of POCD in the patients with cardiac surgery.

Despite improvements in surgical and anesthetic techniques, POCD in cardiac surgery patients is still highly prevalent. Relander et al. reported that POCD was observed in 71% of patients 1 week postoperatively and in 47% of elderly patients 3 months after cardiac surgery [29]. Our study illustrated that the incidence of POCD was 24.4% on postoperative day 7 and 17.1% at 3 months in the patients undergoing thoracoabdominal aortic surgery. The difference for the incidence of POCD in these studies is probably due to the diverse neuropsychological tests and the statistical methodology, as well as the fact that to date there is no universally accepted definition of POCD. Previous studies indicated an association between POD and subsequent POCD in elderly patients undergoing orthopedic surgery [30] and cardiac surgery [31]. A higher incidence and a longer duration of POD were observed in the patients who developed POCD in this study.

There is no internationally accepted definition for POCD. Neuropsychological testing battery is required to evaluate postoperative cognitive function reliably. The precise and inclusive neuropsychological tests were applied in this study, including MMSE, SDMT, DFST, DSBT, RAVLT, SCWT, CAM-ICU, TICS-M and ADLs, which allowed to detect slight changes in cognitive function characteristic of POCD. Consistent with previous studies, the definition of POCD was based on Z-score in this study [21, 23, 24].

The present study demonstrated that elevated level of CSF sTREM2 was correlated with an increased risk of developing POCD in the patients undergoing thoracoabdominal aortic surgery. A growing number of studies demonstrated that CSF sTREM2 was increased in the late-onset AD [32, 33] and early-onset familial AD [34]. The upregulation of CSF sTREM2 was associated with the increased risk of dementia [35]. A study by Tanaka et al. reported that elevated sTREM2 was related to cognitive impairment in non-obese diabetic patients [36]. This study indicated that CSF sTREM2 was associated with cognitive impairment after thoracoabdominal aortic replacement surgery. These findings indicate that elevated CSF sTREM2 may be a potential therapeutic target of POCD.

The underlying mechanism for the relationship between sTREM2 and POCD remains unknown. One explanation is that CSF sTREM2 elevation reflects sTREM2-mediated microglial activation and acute neuroinflammation. Microglial activation and subsequent neuroinflammatory response are frequently accompanied by the early progress of Aβ accumulation, tau pathology, cerebrovascular injury and cognitive impairment [37–41]. A solid relationship between neuroinflammation and POCD has been reported in orthopedic patients, which fully demonstrates the critical role of neuroinflammation in POCD development [42]. Our previous study demonstrated that microglial activation and neuroinflammation involved in the progression of POCD [43]. CSF sTREM2 was increased in AD and considered as a biomarker of microglia activation and neuroinflammation [44, 45]. sTREM2 has been associated with inhibition of the anti-inflammatory function [44]. Similarly, a positive correlation between sTREM2 and the levels of several inflammatory cytokines, suggesting an association between microglial activation and CSF inflammation [46]. Consistent with previous study, this study indicated that POCD development was associated with sTREM2-mediated neuroinflammatory response.

sTREM2 is produced by ectodomain shedding of its receptor, which simultaneously produces peptides that reduce the activity of full-length receptor TREM2 [15]. Our previous study demonstrated that partial hepatectomy downregulated TREM2 expression in APPswe/PS1dE9 mice, coupled with postoperative spatial learning and memory impairment [47]. Thus, the increased level of CSF sTREM2 in the patients with POCD might indirectly reflect that the activity of TREM2 was inhibited following surgery challenge.

Exploring relationship between CSF biomarkers and POCD may provide better understanding of pathogenic mechanism by hinting to simultaneously ongoing processes in the brain [33]. POCD shares similar neuropathological mechanisms with AD. Therefore, multiple studies investigated the association between Aβ_42_, CSF tau or Aβ to tau ratio and POCD [12]. Aβ is generally considered to be associated with POCD [48–50]. A pilot study demonstrated that cognitive decline was related to the reduction of CSF Aβ_42_ in 6 months after cardiac surgery, which is consistent with our study [51]. TREM2 and Aβ precursor protein share common features of ectodomain shedding by ADAMs α-secretase and subsequent γ-secretase cleavage, which suggests the existence of both common biological processes involved in Aβ and sTREM2 [15, 16, 52].

Tau, the main protein component of the neurofibrillary tangles of synapses, has been linked to cognitive dysfunction [9, 53–56]. A study by Freche et al. indicated that sevoflurane exposure increased tau phosphorylation through specific kinases activation and induced spatial memory deficits [57]. Consistent with previous study, the present study demonstrated that surgery and anesthesia-induced accumulation of hyperphosphorylated tau proteins was associated with cognitive impairment. However, a study by Berger et al. failed to reveal the significant change of CSF tau, P-tau-181p, or Aβ levels before and after surgery in non-cardiac surgery [58]. This is contrary to our findings, which may be due to the various time points of assessment, the different definition of POCD, as well as the multiple types of surgery. Xie et at demonstrated that preoperative CSF Aβ/tau ratio was associated with postoperative changes in specific cognitive domains [11]. In line with this, lower postoperative CSF Aβ_42_/tau ratio was observed in patients with POCD in this study. Thus, a combination of CSF sTREM2 and other POCD biomarkers may provide reliable and convenient predictor of POCD.

There are some limitations in this study. Firstly, the sample size is relatively small and more large-scaled multicenter studies are required to determine the predictive value of sTREM2 for POCD. Secondly, the sample of CSF was not obtained conveniently and CSF sTREM2 could not measure routinely in the clinical practice. Thirdly, this study only investigated short-term follow-up outcome. Longer-term follow-up is needed to validate the role of sTREM2 in the progression of POCD. Intubated patients were excluded on postoperative days 3 and 7 in this study. However, CAM-ICU is insensitive for delirium in non-intubated patients. A large fraction of delirium cases might be missed in this study. Finally, the intrathecal catheters were removed on postoperative day 3. However, this experiment could not totally exclude intrathecal catheters-induced neuroinflammation.

In conclusion, the protein level of CSF sTREM2 was significantly increased following surgery and anesthesia. Anesthesia and surgery-induced elevation of CSF sTREM2 is associated with an increased risk of early cognitive dysfunction following surgery. This finding may provide valuable mechanistic insights into the etiology of POCD. Thus, a combination of CSF sTREM2 and other POCD predictors may be reliable to identify high-risk persons of early cognitive dysfunction following surgery.

## References

[1] Kotekar N, Shenkar A, Nagaraj R (2018). Postoperative cognitive dysfunction - current preventive strategies. Clin Interv Aging.

[2] Newman M, Kirchner J, Phillips-Bute B, Gaver V, Grocott H, Jones R (2001). Longitudinal assessment of neurocognitive function after coronary-artery bypass surgery. N Engl J Med.

[3] Hogue C, Gottesman R, Stearns J (2008). Mechanisms of cerebral injury from cardiac surgery. Crit Care Clin.

[4] Monk T, Weldon B, Garvan C, Dede D, van der Aa M, Heilman K (2008). Predictors of cognitive dysfunction after major noncardiac surgery. Anesthesiology.

[5] Jacob Steinmetz KBC, Lund T, Lohse N, Lars S (2009). Rasmussen and the ISPOCD group: long term consequences of postoperative cognitive dysfunction. Anesthesiology.

[6] Czyz-Szypenbejl K, Medrzycka-Dabrowska W, Kwiecien-Jagus K, Lewandowska K (2019). The occurrence of postoperative cognitive dysfunction (POCD) - systematic review. Psychiatr Pol.

[7] Rasmussen L, Christiansen M, Rasmussen H, Kristensen P, Moller J (2000). Do blood concentrations of neurone specific enolase and S-100 beta protein reflect cognitive dysfunction after abdominal surgery?ISPOCD Group. Br J Anaesth.

[8] Linstedt U, Meyer O, Kropp P, Berkau A, Tapp E, Zenz M (2002). Serum concentration of S-100 protein in assessment of cognitive dysfunction after general anesthesia in different types of surgery. Acta Anaesthesiol Scand.

[9] Wiberg S, Holmgaard F, Zetterberg H, Nilsson J, Kjaergaard J, Wanscher M (2021). Biomarkers of cerebral injury for prediction of postoperative cognitive dysfunction in patients undergoing cardiac surgery. J Cardiothorac Vasc Anesth.

[10] Ramlawi B, Rudolph JL, Mieno S, Khabbaz K, Sodha NR, Boodhwani M, Levkoff SE, Marcantonio ER, Sellke FW (2006). Serologic markers of brain injury and cognitive function after cardiopulmonary bypass. Ann Surg.

[11] Xie Z, McAuliffe S, Swain C, Ward S, Crosby C, Zheng H (2013). Cerebrospinal fluid aβ to tau ratio and postoperative cognitive change. Ann Surg.

[12] Wu Z, Zhang M, Zhang Z, Dong W, Wang Q, Ren J (2018). Ratio of beta-amyloid protein (Abeta) and tau predicts the postoperative cognitive dysfunction on patients undergoing total hip/knee replacement surgery. Exp Ther Med.

[13] Zhong J, Li J, Ni C, Zuo Z (2020). Amantadine alleviates postoperative cognitive dysfunction possibly by preserving neurotrophic factor expression and dendritic Arborization in the Hippocampus of old rodents. Front Aging Neurosci.

[14] Jiang T, Yu JT, Zhu XC, Tan L (2013). TREM2 in Alzheimer's disease. Mol Neurobiol.

[15] Wunderlich P, Glebov K, Kemmerling N, Tien N, Neumann H, Walter J (2013). Sequential proteolytic processing of the triggering receptor expressed on myeloid cells-2 (TREM2) protein by ectodomain shedding and γ-secretase-dependent intramembranous cleavage. J Biol Chem.

[16] Kleinberger G, Yamanishi Y, Suarez-Calvet M, Czirr E, Lohmann E, Cuyvers E (2014). TREM2 mutations implicated in neurodegeneration impair cell surface transport and phagocytosis. Sci Transl Med.

[17] Steiner A, Schlepckow K, Brunner B, Steiner H, Haass C, Hagn F (2020). γ-Secretase cleavage of the Alzheimer risk factor TREM2 is determined by its intrinsic structural dynamics. EMBO J.

[18] Ewers M, Franzmeier N, Suárez-Calvet M, Morenas-Rodriguez E, Caballero M, Kleinberger G (2019). Increased soluble TREM2 in cerebrospinal fluid is associated with reduced cognitive and clinical decline in Alzheimer's disease. Sci Transl Med.

[19] Zhong L, Chen X (2019). The emerging roles and therapeutic potential of soluble TREM2 in Alzheimer's disease. Front Aging Neurosci.

[20] Ferri E, Rossi PD, Geraci A, Ciccone S, Cesari M, Arosio B (2020). The sTREM2 concentrations in the blood: a marker of neurodegeneration?. Front Mol Biosci.

[21] Krenk L, Kehlet H, Baek Hansen T, Solgaard S, Soballe K, Rasmussen LS (2014). Cognitive dysfunction after fast-track hip and knee replacement. Anesth Analg.

[22] Nassiri F, Workewych AM, Badhiwala JH, Cusimano MD (2018). Cognitive outcomes after anterior communicating artery aneurysm repair. Can J Neurol Sci.

[23] Han Y, Han L, Dong MM, Sun QC, Zhang ZF, Ding K (2019). Preoperative salivary cortisol AM/PM ratio predicts early postoperative cognitive dysfunction after noncardiac surgery in elderly patients. Anesth Analg.

[24] Wan J, Luo P, Du X, Yan H (2020). Preoperative red cell distribution width predicts postoperative cognitive dysfunction after coronary artery bypass grafting. Biosci Rep.

[25] Moller JT, Cluitmans P, Rasmussen LS, Houx P, Rasmussen H, Canet J (1998). Long-term postoperative cognitive dysfunction in the elderly ISPOCD1 study. ISPOCD investigators. International study of post-operative cognitive dysfunction. Lancet.

[26] Qiao Y, Feng H, Zhao T, Yan H, Zhang H, Zhao X (2015). Postoperative cognitive dysfunction after inhalational anesthesia in elderly patients undergoing major surgery: the influence of anesthetic technique, cerebral injury and systemic inflammation. BMC Anesthesiol.

[27] Silva FP, Schmidt AP, Valentin LS, Pinto KO, Zeferino SP, Oses JP (2016). S100B protein and neuron-specific enolase as predictors of cognitive dysfunction after coronary artery bypass graft surgery: a prospective observational study. Eur J Anaesthesiol.

[28] Ryan SL, Kimchi EY (2021). Evaluation and Management of Delirium. Semin Neurol.

[29] Relander K, Hietanen M, Rantanen K, Ramo J, Vento A, Saastamoinen KP (2020). Postoperative cognitive change after cardiac surgery predicts long-term cognitive outcome. Brain Behav.

[30] Bickel H, Gradinger R, Kochs E, Förstl H (2008). High risk of cognitive and functional decline after postoperative delirium. A three-year prospective study. Dement Geriatr Cogn Disord.

[31] Saczynski J, Marcantonio E, Quach L, Fong T, Gross A, Inouye S (2012). Cognitive trajectories after postoperative delirium. N Engl J Med.

[32] Heslegrave A, Heywood W, Paterson R, Magdalinou N, Svensson J, Johansson P. Increased cerebrospinal fluid soluble TREM2 concentration in Alzheimer's disease. Mol Neurodegener. 2016;11:3. 10.1186/s13024-016-0071-x PMC4709982 26754172

[33] Henjum K, Quist-Paulsen E, Zetterberg H, Blennow K, Nilsson LNG, Watne LO (2018). CSF sTREM2 in delirium-relation to Alzheimer's disease CSF biomarkers Abeta42, t-tau and p-tau. J Neuroinflammation.

[34] Suárez-Calvet M, Kleinberger G, Araque Caballero M, Brendel M, Rominger A, Alcolea D (2016). sTREM2 cerebrospinal fluid levels are a potential biomarker for microglia activity in early-stage Alzheimer's disease and associate with neuronal injury markers. EMBO Mol Med.

[35] van der Ende E, Morenas-Rodriguez E, McMillan C, Grossman M, Irwin D, Sanchez-Valle R (2021). CSF sTREM2 is elevated in a subset in GRN-related frontotemporal dementia. Neurobiol Aging.

[36] Tanaka M, Yamakage H, Masuda S, Inoue T, Ohue-Kitano R, Araki R (2019). Serum soluble TREM2 is a potential novel biomarker of cognitive impairment in Japanese non-obese patients with diabetes. Diabetes Metab.

[37] Mosher K, Wyss-Coray T (2014). Microglial dysfunction in brain aging and Alzheimer's disease. Biochem Pharmacol.

[38] Streit W, Xue Q, Tischer J, Bechmann I (2014). Microglial pathology. Acta Neuropathol Commun.

[39] Heneka M, Carson M, El Khoury J, Landreth G, Brosseron F, Feinstein D (2015). Neuroinflammation in Alzheimer's disease. Lancet Neurol.

[40] Tanzi R (2015). TREM2 and Risk of Alzheimer's Disease--Friend or Foe?. N Engl J Med.

[41] Cattaneo A, Cattane N, Galluzzi S, Provasi S, Lopizzo N, Festari C (2017). Association of brain amyloidosis with pro-inflammatory gut bacterial taxa and peripheral inflammation markers in cognitively impaired elderly. Neurobiol Aging.

[42] Cho I, Kim J, Kim E, Kim S, Kam E, Cheong E (2021). Orthopedic surgery-induced cognitive dysfunction is mediated by CX3CL1/R1 signaling. J Neuroinflammation.

[43] Cao X, Ma H, Wang J, Liu F, Wu B, Tian A (2010). Postoperative cognitive deficits and neuroinflammation in the hippocampus triggered by surgical trauma are exacerbated in aged rats. Prog Neuro-Psychopharmacol Biol Psychiatry.

[44] Piccio L, Buonsanti C, Cella M, Tassi I, Schmidt R, Fenoglio C (2008). Identification of soluble TREM-2 in the cerebrospinal fluid and its association with multiple sclerosis and CNS inflammation. Brain.

[45] Wilson EN, Swarovski MS, Linortner P, Shahid M, Zuckerman AJ, Wang Q (2020). Soluble TREM2 is elevated in Parkinson's disease subgroups with increased CSF tau. Brain.

[46] Azzolini F, Gilio L, Pavone L, Iezzi E, Dolcetti E, Bruno A, et al. Neuroinflammation is associated with GFAP and sTREM2 levels in multiple sclerosis. Biomolecules. 2022;12(2):222. 10.3390/biom12020222 PMC8961656 35204724

[47] Jiang Y, Li Z, Ma H, Cao X, Liu F, Tian A (2018). Upregulation of TREM2 ameliorates Neuroinflammatory responses and improves cognitive deficits triggered by surgical trauma in Appswe/PS1dE9 mice. Cell Physiol Biochem.

[48] Zuo C, Wang C, Liu J, Shen T, Zhou J, Hao X (2018). Isoflurane anesthesia in aged mice and effects of A1 adenosine receptors on cognitive impairment. CNS Neurosci Ther.

[49] Zhang J, Zhu S, Jin P, Huang Y, Dai Q, Zhu Q (2020). Graphene oxide improves postoperative cognitive dysfunction by maximally alleviating amyloid beta burden in mice. Theranostics.

[50] Kim J, Jung H, Lee Y, Sohn J (2021). Surgery performed under Propofol anesthesia induces cognitive impairment and amyloid pathology in ApoE4 Knock-in mouse model. Front Aging Neurosci.

[51] Palotas A, Reis HJ, Bogats G, Babik B, Racsmany M, Engvau L (2010). Coronary artery bypass surgery provokes Alzheimer's disease-like changes in the cerebrospinal fluid. J Alzheimers Dis.

[52] O'Brien R, Wong P (2011). Amyloid precursor protein processing and Alzheimer's disease. Annu Rev Neurosci.

[53] Xie Z, Culley D, Dong Y, Zhang G, Zhang B, Moir R (2008). The common inhalation anesthetic isoflurane induces caspase activation and increases amyloid beta-protein level in vivo. Ann Neurol.

[54] Bissette G (2009). Mini-forum: roles of amyloid-beta and tau phosphorylation in neuronal repair and protection. J Alzheimers Dis.

[55] Wang Y, Lv J, He J, Wen G, Wu X (2022). Mechanism of psychoactive substance-induced cognitive disorders: does tau protein play a role?. Front Biosci (Landmark Ed).

[56] Shi HJXX, Wang YL, Zhang WS, Wang ZS, Yu AL (2015). Effects of different anesthesia methods on cognitive dysfunction after hip replacement operation in elder patients. Int J Clin Exp Med.

[57] Le Freche HBJ, Fernandez-Gomez FJ, Patin P, Caillierez R, Zommer N, Sergeant N, Buée-Scherrer V, Lebuffe G, Blum D, Buée L (2012). Tau phosphorylation and sevoflurane anesthesia: an association to postoperative cognitive impairment. Anesthesiology.

[58] Berger M, Browndyke J, Cooter Wright M, Nobuhara C, Reese M, Acker L, Bullock W, Colin B, Devinney M, Moretti E (2022). Postoperative changes in cognition and cerebrospinal fluid neurodegenerative disease biomarkers. Ann Clin Transl Neurol.

